# Down‐regulation of microRNAs of the *miR‐200* family and up‐regulation of Snail and Slug in inflammatory bowel diseases — hallmark of epithelial−mesenchymal transition

**DOI:** 10.1111/jcmm.12869

**Published:** 2016-04-26

**Authors:** Nina Zidar, Emanuela Boštjančič, Miha Jerala, Nika Kojc, David Drobne, Borut Štabuc, Damjan Glavač

**Affiliations:** ^1^ Institute of PathologyFaculty of MedicineUniversity of LjubljanaLjubljanaSlovenia; ^2^ Department of GastroenterologyUniversity Medical Centre LjubljanaLjubljanaSlovenia

**Keywords:** Crohn's disease, ulcerative colitis, fibrosis, epithelial−mesenchymal transition, microRNA, Snail, Slug

## Abstract

Fibrosis is an important feature of inflammatory bowel diseases (IBD), particularly Crohn's disease (CD), but its pathogenesis is poorly understood. To determine the postulated involvement of epithelial−mesenchymal transition (EMT) in the development of fibrosis in IBD, we analysed the expression profiles of the *miR‐200* family which has been shown to induce EMT in experimental models and various human diseases. We also analysed the expression of Snail and Slug, postulated targets of the investigated microRNAs. Ten patients with ulcerative colitis (UC) and 10 patients with CD who underwent colon resection were included. From each, two tissue samples were chosen (one with the most severely and one with the least affected or normal mucosa) for analysis of microRNAs expression using real‐time polymerase chain reaction, and Snail and Slug expression using immunohistochemistry. We found significant down‐regulation of all investigated microRNAs in CD, and of three investigated microRNAs in UC, in comparison to the normal or the least affected mucosa. Comparing UC and CD, four microRNAs were significantly more down‐regulated in CD than in UC. Snail and Slug were expressed in the injured epithelium and occasionally in mesothelial cells and submesothelial fibroblasts. Our finding of down‐regulation of the *miR‐200* family and up‐regulation of transcription repressors Snail and Slug supports the postulated role of EMT in the pathogenesis of fibrosis in IBD. The described expression patterns are consistent with the notion that fibrosis does not occur only in CD but also in UC, being much more severe in CD.

## Introduction

Fibrosis is an important feature of inflammatory bowel diseases (IBD), with serious clinical consequences. There are significant differences between Crohn's disease (CD) and ulcerative colitis (UC). In CD, inflammation and fibrosis are usually transmural, often leading to stenosis and stricture formation 1. Stricture formation necessitating surgery will occur in up to 50% of patients with CD within 10 years of disease onset 2. Moreover, stricture commonly recurs at surgical anastomosis 3, requiring multiple surgeries. In UC, on the contrary, inflammation and fibrosis are restricted to mucosa and submucosa. Fibrosis may contribute to shortening and stiffening of the colon, but rarely results in clinically important stricture formation 4.

A significant progress has been made in the treatment of IBD, mainly targeting inflammation. In contrast, little progress has been made in the treatment of fibrosis 5. Cosnes *et al*. 6 reported an unchanged incidence of strictures and need for surgery during the last 25 years despite the more frequent use of immunosuppressive therapy. There is, therefore, an urgent need to develop new treatment modalities to prevent stricture.

One of the reasons for poor success in the treatment of fibrosis in IBD is that its pathogenesis is poorly understood. Similar to fibrosis in other diseases, it is believed to result from tissue damage due to chronic inflammation and impaired mechanisms of wound healing 7, 8. Fibrosis is characterized by proliferation of activated myofibroblasts and an excessive deposition of extracellular matrix proteins. The mechanisms by which inflammation leads to fibrosis are only beginning to be understood, but most likely involve epithelial−mesenchymal transition (EMT) 9. EMT is believed to be a key contributor to the pool of activated fibroblasts in fibrosis in various organs, such as the kidney, lung and liver 7, 10. There are limited data regarding EMT in IBD.

MicroRNAs are small, non‐coding RNAs that regulate gene expression by post‐transcriptional regulation of target genes. Due to incomplete complementarity, binding of miRNA to target 3′‐UTR causes the absence of protein synthesis by inhibition of translation of its mRNA 11. They are involved in a variety of physiological functions as well as in various human diseases including IBD 12, 13.

To analyse the postulated involvement of EMT in the development of fibrosis in IBD, we analysed the expression profiles of microRNAs of the *miR‐200* family which have been shown in previous studies to induce EMT in experimental models and in human diseases 14, 15, 16, 17. We also analysed the expression of Snail and Slug, the postulated targets of the investigated miRNAs 14, 18, 19.

## Materials and methods

### Patients

Our study, which was approved by the Institute's Review Board, included 40 tissue samples from 20 patients who underwent colon resection (10 patients with UC, 10 patients with CD). The diagnosis of IBD was made on the basis of clinical, radiological, endoscopic and histological findings 20, 21, 22. For the purpose of this study, the most important demographic and clinical data were collected (IBD type, duration and maximal extent of the disease, therapy, indication for surgery).

Resection specimens had been handled according to standard procedures 22. Samples had been taken from inflamed mucosa and ulcers as well as from macroscopically normal mucosa. All samples had been embedded in paraffin and cut at 4 μm, stained with haematoxylin and eosin and analysed according to standard criteria 22.

For the purpose of this study, all slides were reviewed. Two slides with corresponding paraffin blocks were chosen from each case for immunohistochemistry and quantitative real‐time PCR (qPCR): one with most severe fibrosis upon histological examination and one from the normal mucosa, if present, or from the least affected mucosa, if normal mucosa was not present in the resection specimen. It may have contained mild inflammation, but not erosions, ulcers or fibrosis.

### Immunohistochemistry

For immunohistochemistry, we used antibodies against Snail+Slug (dilution 1:200, ab85936; Abcam, Cambridge, UK) and E‐cadherin (dilution 1:20, SPM471; Labvision, Fremont, CA, USA). Sections were treated with biotinylated secondary antibody, followed by incubation with peroxidase conjugated streptavidin (iVIEW^™^ DAB Detection Kit; Ventana Medical System, Tucson, AZ, USA). Visualization of the immunoreaction was carried out with 3.3′ diaminobenzidine. Finally, sections were counterstained with haematoxylin. Adult fibrosarcoma served as a positive control for Snail and Slug, and oral mucosa for E‐cadherin. Positive controls and negative controls omitting the primary antibodies were also included.

### RNA isolation

Tissue samples were cut at 10 μm from formalin‐fixed paraffin‐embedded tissue blocks using a microtome. Six to eight 10‐μm sections were used for the isolation procedure. Total RNA isolation was performed with a miRNeasy FFPE kit (Qiagen, Hilden, Germany) according to the manufacturer's protocol. All the reagents were from Qiagen, except where otherwise indicated. Briefly, 1 ml of Xylene (Merck, Kenilworth, NJ, USA) was added for de‐paraffinization, followed by brief vortexing and centrifugation. After the ethanol‐washing step, pellets were air‐dried and digestion with proteinase K was performed at 55°C for 15 min, followed by 15 min incubation at 80°C in order to partially reverse formaldehyde modification of nucleic acid. After the gDNA elimination step, 100% ethanol (Merck) was added to the samples and the mixture was transferred to an RNeasy MiniElute spin column. After two washing steps, the RNA was eluted in 30 μl of nuclease‐free water. The concentration was measured NanoDrop‐1000.

### Quantitative real‐time PCR

Looped primers for specific reverse transcription (RT) of miRNAs were utilized following the manufacturer's protocol. RNU6B was used as RG. MicroRNAs, *miR‐141*,* miR‐200a*,* miR‐200b*,* miR‐200c* and *miR‐429* were tested relatively to RNU6B. Briefly, 10 μl RT reaction master mix was performed with 10 ng of total RNA sample, 1.0 μl of MultiScribe Reverse Transcriptase (50 U/μl), 1 μl of RT Buffer (10×), 0.1 μl of dNTP (100 mM), 0.19 μl RNAase inhibitor (20 U/μl) and 2 μl of RT primer (5×). The reaction conditions were: at 16°C for 30 min, at 42°C for 30 min, at 85°C for 5 min.

Quantitative real‐time PCR was carried out in 20 μl PCR master mix containing 5 μl TaqMan 2× Universal PCR Master Mix, 0.5 μl TaqMan assay and 4.5 μl RT products diluted 16‐fold. The qPCR reactions were performed in duplicates as following: initial denaturation at 95°C for 10 min, and 40 cycles for 15 s at 95°C (denaturation), for 60 s at 60°C (primers annealing and elongation). The signal was collected at the end‐point of every cycle.

Prior to qPCR analysis, four pools of RNA samples were created, obtained from UC and corresponding normal mucosa, and from CD and corresponding normal mucosa. After RT, the cDNA was diluted in five steps, ranging from 4‐point dilution to 1024‐point dilution, and the probes were tested for qPCR efficiency. All the qPCR efficiency reactions were performed in triplicate.

### Statistical analysis

To present relative gene expression, the method referred as the 2^−ΔΔCt^ corrected for PCR efficiencies was used 23. Relative gene expression presents the data of the gene of interest (GOI, Ct_GOI_) relative to reference gene (RG, Ct_RG_), named ΔCt. Calculated ΔCt of CD or UC and adjunct normal mucosa were compared to and tested for statistical significance. For comparison of CD and UC to each other, samples were normalized to corresponding normal mucosa to obtain ΔΔCt and tested for difference in expression.

For the calculation of statistical differences, the SPSS analytical software ver. 20, (SPSS Inc., Chicago, IL, USA) was used, more precisely two‐tailed with a cut‐off point at *P* < 0.05. Mann–Whitney test was used for comparison of UC and CD. Wilcoxon Rank test was used for comparison of UC or CD to corresponding normal mucosa. The data were presented as fold change in graph with error bars presenting calculated fold change error using SD of the Ct triplicates.

## Results

The most important demographic and clinical data at the time of surgery are presented in Table 1. Among patients with UC, there were four men and six women, aged 16–70 years (50.6 ± 18.3). Among patients with CD, there were four men and six women, aged 25–59 years (41.7 ± 13.1). The duration of the disease from the initial diagnosis to surgery ranged from 1 month to 15 years for UC and from 1 month to 35 years for CD. The most frequent indication for resection was stenosis in patients with CD and poor response to therapy in patients with UC. Normal mucosa was available in four patients with UC and in six patients with CD. In the remaining six patients with UC and four patients with CD, samples from the least affected mucosa were included as controls.

**Table 1:** The most important demographic and clinical data of patients with inflammatory bowel diseases

	Ulcerative colitis (*n* = 10)	Crohn's disease (*n* = 10)
Male:female	2:3	2:3
Age (years) (mean ± SD)	50.6 ± 18.28	41.7 ± 13.1
Duration of disease (years) (mean ± SD)	7.3 ± 5.1	11.9 ± 10.7
Indication for surgery		
Treatment failure	10 (100)	2 (20)
Perforation	0	1 (10)
Stenosis	0	6 (60)
Fistulas	0	1 (10)
Extent of disease		
Ileum	0	0
Colon	8 (80)	5 (50)
Ileum and colon	0	5 (50)
Rectosigmoid	2 (40)	0
Medication		
Methotrexate	0	2 (20)
Infliximab	6 (60)	5 (50)
Azathioprine	7 (70)	6 (60)
Mycophenolate mofetil	0	1 (10)
Adalimumab	3 (30)	4 (40)

Values in parentheses are percentages.

### Expression of the miR‐200 family in ulcerative colitis and Crohn's disease compared to corresponding normal mucosa

**Figure 1:**
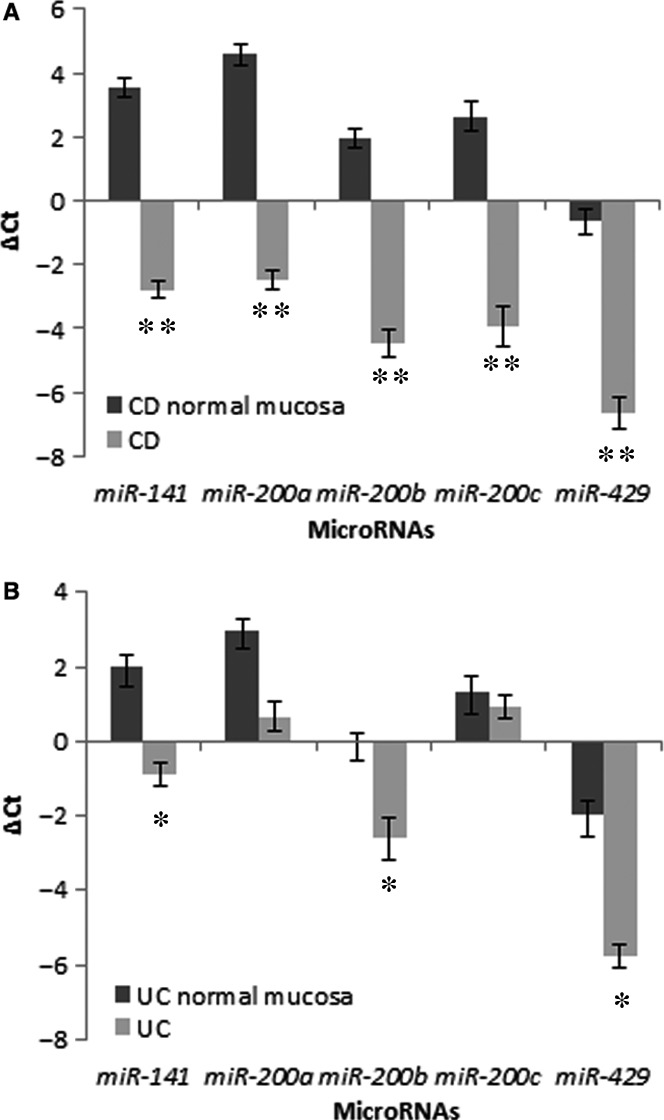
Expression of microRNAs of the miR‐200 family in Crohn's disease (**A**) and ulcerative colitis (**B**) in comparison to the corresponding normal mucosa. CD, Crohn's disease; UC, ulcerative colitis; *P ≤ 0.05; **P ≤ 0.01.

MicroRNAs *miR‐141*,* miR‐200a*,* miR‐200b*,* miR‐200c* and *miR‐429* (ΔCt analysis) were down‐regulated in CD (~80.1‐fold, *P* = 0.010; ~131.9‐fold, *P* = 0.008; ~86.0‐fold, *P* = 0.008; ~96.9‐fold, *P* = 0.010; and ~65.0‐fold, *P* = 0.010, respectively; Wilcoxon signed ranks test) when compared to corresponding normal or the least affected mucosa. The results are summarized in Fig. 1A.

Similarly, microRNAs *miR‐141*,* miR‐200b* and *miR‐429* were down‐regulated in UC (~7.5‐fold, *P* = 0.028; ~5.9‐fold, *P* = 0.047; and ~13.8‐fold, *P* = 0.022 respectively; Wilcoxon signed ranks test) when compared to corresponding normal mucosa. In contrast to CD, *miR‐200a* down‐regulation (~4.9‐fold) did not reach statistical significance, whereas expression of *miR‐200c* was similar as in corresponding normal or the least affected mucosa. The results are summarized in Fig. 1B.

### Expression of the *miR‐200* family in ulcerative colitis compared to Crohn's disease

**Figure 2:**
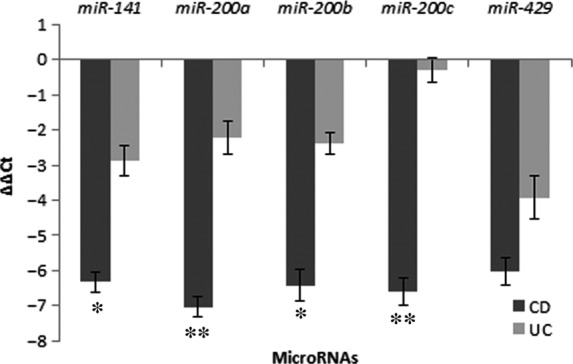
Expression of microRNAs of the miR‐200 family in Crohn's disease in comparison to ulcerative colitis. CD, Crohn's disease; UC, ulcerative colitis; *P < 0.05; **P ≤ 0.01.

Four of five investigated miRNAs, *miR‐141*,* miR‐200a*,* miR‐200b* and *miR‐200c*, were significantly down‐regulated in CD (ΔΔCt analysis) in comparison to UC (*P* = 0.041, *P* = 0.009, *P* = 0.041 and *P* = 0.007 respectively; Mann–Whitney test). Expression of *miR‐429* was also down‐regulated but did not reach statistical significance. The results are summarized in Fig. 2.

### Immunohistochemical expression of Snail, Slug and E‐cadherin

**Figure 3:**
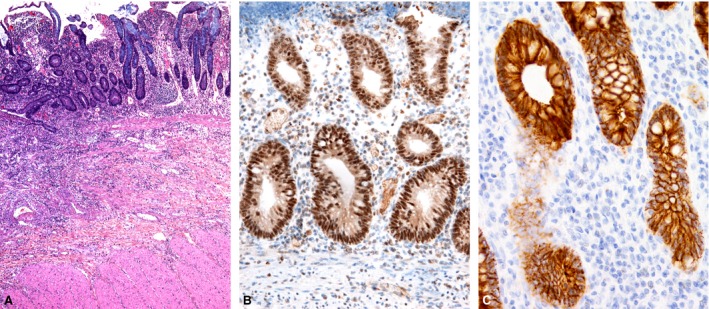
Crohn's disease. (**A**) Distorted crypt architecture, inflammation in the lamina propria and submucosa. (**B**) Immunohistochemistry for Snail and Slug: positive nuclear and cytoplasmic reaction for Snail and Slug in the crypt epithelium and some inflammatory cells. (**C**) Immunohistochemistry for E‐cadherin: positive membraneous reaction in the crypt epithelium, with a focal decreased intensity of staining.

**Figure 4:**
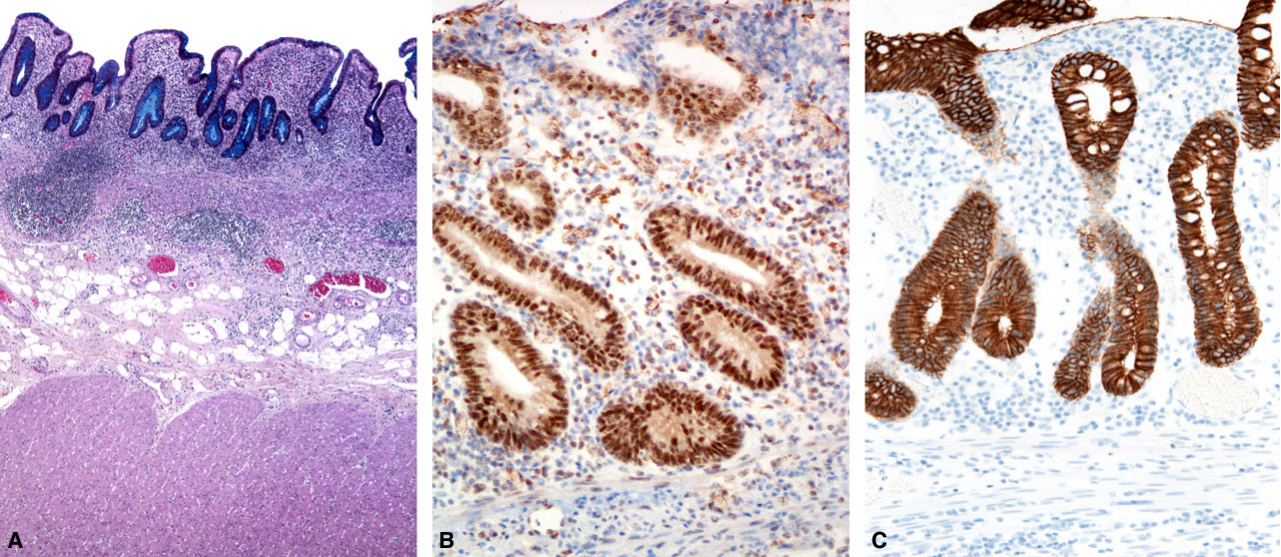
Ulcerative colitis. (**A**) Distorted crypt architecture, inflammation in the lamina propria and in the upper part of submucosa. (**B**) Immunohistochemistry for Snail and Slug: positive nuclear and cytoplasmic reaction for Snail and Slug in the crypt epithelium and some inflammatory cells. (**C**) Immunohistochemistry for E‐cadherin: positive membranous reaction in the crypt epithelium, with a focal decreased intensity of staining.

**Figure 5:**
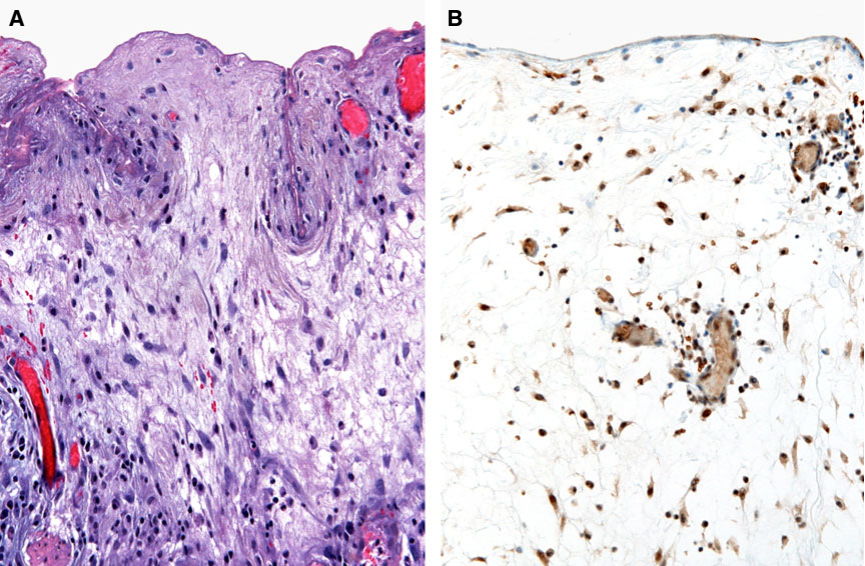
Crohn's disease. (**A**) Proliferation of myofibroblasts in the subserosal layer, with a positive immunohistochemistry for Snail and Slug (**B**).

Snail and Slug expression was found in the crypt epithelium in 10 cases of CD (Fig. 3B) and in seven cases of UC (Fig. 4B), reaction was nuclear and cytoplasmic. They were also expressed in the mesothelial cells and in subserosal fibroblasts (Fig. 5B) in six and three cases of CD and UC respectively. The differences between CD and UC were not statistically significant.

In the normal mucosa, Snail and Slug were expressed in the nerve fibres, ganglion cells and in erythrocytes. No positivity was found in the epithelium, fibroblasts, mesothelial cells or smooth muscle cells.

Immunohistochemistry for E‐cadherin showed a decreased intensity of staining in areas of crypt destruction and cryptitis. In the preserved crypts, it exhibited almost a diffuse membranous reaction. There was a focal decrease in staining at the base and neck of the crypts (Figs 3C and 4C) in all cases.

## Discussion

In this study, we analysed microRNAs of the *miR‐200* family in UC and CD. The *miR‐200* family comprises *miR‐200a/b/c*,* miR‐141* and *miR‐429* and has been shown to induce EMT in experimental models and in various human diseases 14, 15, 24. We found significant down‐regulation of the investigated microRNAs in both UC and CD in comparison to the normal or the least affected mucosa. These findings support the hypothesis that EMT does occur in IBD, probably contributing to the development of fibrosis, and that this is, at least partly, controlled by microRNAs of the miR‐200 family.

Comparison of microRNAs of the *miR‐200* family showed that all miRNAs were down‐regulated in CD, whereas only three of five were down‐regulated in UC. It has been suggested that miRNAs from the same family may target the same process through co‐operativity to obtain more effective regulation 25. Speculatively, down‐regulation of all five miRNAs in CD might give a more efficient EMT induction leading to a more profound fibrosis compared to only three of five miRNAs down‐regulated in UC.

When we compared CD and UC, we found that all miRNAs were more down‐regulated in CD than in UC. Further comparison of miRNAs expression in CD and UC revealed that the expression of four of five investigated miRNAs (*miR‐141*,* miR‐200a*,* miR‐200b* and *miR‐200c*) was significantly lower in CD than in UC. This microRNA expression pattern is consistent with the notion that fibrosis does not occur only in CD but also in UC 4, 26, being much more common and severe in CD. The reported prevalence of clinically important fibrosis and strictures ranges from 30% to 50% for CD 27 and from 1% to 11% for UC, depending largely on its definition 4, 28. The difference between CD and UC is not surprising, as fibrosis in CD is mostly transmural and in UC, it mostly affects mucosa and submucosa, and only occasionally extends to deeper layers of the bowel wall. This pattern of fibrosis in IBD was also confirmed in our study. The possible explanation would be that *miR‐200* family might regulate different target genes under different pathophysiological conditions 15.

The investigated microRNAs have been demonstrated in previous studies of various human diseases and experimental models to be strongly associated with EMT 14, 15, 16, 17. The significance of EMT in the pathogenesis of fibrosis in IBD is controversial. It likely contributes to the pool of myofibroblasts which are the key cells in the development of fibrosis. Once activated, they produce and secrete excessive amounts of extracellular matrix proteins, such as collagens, fibronectin and tenascin. They also exhibit increased proliferation and decreased apoptosis, and contribute to the self‐perpetuating process of fibrosis 27.

The key role of myofibroblasts in fibrogenesis in various diseases is well accepted 7. However, the source of these important cells remains controversial, particularly regarding the injured epithelium as their possible origin. There is mounting evidence that myofibroblasts may originate from the injured epithelium *via* EMT in various diseases of the kidney, lung, liver and other organs 7, 29, 30. The information about IBD is scarce, but in a mouse model of CD, Flier *et al*. 9 demonstrated that approximately one‐third of myofibroblasts were derived from intestinal epithelial cells through EMT. Some authors, on the contrary, believe that EMT does not contribute to the formation of myofibroblasts *in vivo*, and suggest that resident mesenchymal cells, *e.g*. fibrocytes and perivascular pericytes and not epithelial cells are the primary source of myofibroblasts in the kidney and other organs 31, 32, 33. However, even in these models, the injured epithelium seems to contribute to myofibroblast formation in a paracrine mode, through various ligands, cytokines and chemokines 31, 33. Both explanations seem acceptable regarding our results. According to one, the injured intestinal epithelial cells are a direct source of myofibroblasts *via* EMT, and according to the other, the EMT signalling deriving from the injured epithelium induces myofibroblast formation from resident mesenchymal cells (*e.g*. fibroblasts and pericytes) by paracrine cell signalling rather than cellular transition.

To further test the hypothesis that EMT is induced in IBD, we analysed the expression of markers of EMT — Snail and Slug which are believed to be regulated by the investigated microRNAs. We found overexpression of Snail and Slug in the injured crypt epithelium in both CD and UC and occasionally in mesothelial cell and subserosal fibroblasts, particularly in CD with severe transmural fibrosis. This finding indicates that in addition to the crypt epithelium, activation of the mesothelial cells and subserosal fibroblasts in cases with transmural inflammation might also be important in the development of fibrosis.

Snail and Slug mediate transcription repression which has emerged as a fundamental mechanism for induction of EMT. In experimental models, Snail, Slug and other transcription repressors induce a complete EMT at both morphological and behavioural levels. The exact mechanisms of transcription repressors have not been completely elucidated, but most likely include down‐regulation of E‐cadherin transcription, resulting in a functional loss of E‐cadherin 34, 35. Immunohistochemistry for E‐cadherin in our study showed only a focal decrease in staining of the crypt epithelium in all cases and is therefore not useful to confirm the proposed effect of transcription repression on intestinal mucosa.

MicroRNAs have emerged as important regulators of various human diseases including IBD 36, 37, 38. Previous studies confirmed that miRNA expression is dysregulated in intestinal mucosa and serum of patients with IBD 12, 39, especially microRNAs that play a role in the regulation of innate and adaptive immune response 12. Few studies have also focused on the role of microRNAs in the development of fibrosis and stenosis, particularly in CD, and presented some promising results. MicroRNAs of the *miR‐29* family have been found to be down‐regulated in the mucosa overlying strictured areas in comparison to adjacent non‐strictured areas in CD 40. Lower serum levels of *miR‐19* were found in CD patients with strictures in comparison to CD patients without strictures, suggesting that *miR‐19* is a promising circulating biomarker of stricturing CD 39. Chen *et al*. 17, 41 reported on a decreased expression of *miR‐200b* in inflamed mucosa and overexpression of *miR‐200b* in the serum from patients with IBD, associated with a decreased expression of E‐cadherin and increased expression of vimentin. They also found that ZEB1, a transcription repressor of E‐cadherin and a direct target gene of *miR‐200b*, was decreased by miR‐200b. They concluded that *miR‐200b* plays a role in maintaining intact intestinal epithelium through inhibiting EMT.

There are several limitations in our study. The most important one is related to the selection criteria. Only patients who underwent surgery were included, resulting in a selection bias, with only the most severe cases of IBD being analysed. It is also not possible to exclude the effect of duration of the disease and treatment, and the effect of the treatment modalities on the histological, genetic and immunohistochemical features. Surgical resection specimens are more suitable for studying fibrosis in IBD, as the whole thickness of the bowel wall is needed, but in routine diagnostic work, we are dealing with endoscopic biopsies. To overcome these problems, future studies should focus on the expression of the investigated microRNAs in the serum and in endoscopic biopsies, to see whether they could be used as a marker of fibrosis.

## Conclusion

Our finding of down‐regulation of microRNAs of the *miR‐200* family supports the postulated role of EMT in the pathogenesis of fibrosis in UC and CD. These microRNAs act on transcription repressors which have been demonstrated to be up‐regulated in the analysed cases. These results add to our understanding of the pathogenetic mechanisms of fibrosis in IBD, which will hopefully enable to develop new treatment modalities. Furthermore, it is hoped that some of them will prove useful as markers to detect early in the course of the disease in patients who are at risk to develop severe fibrosis 42.

## Conflict of interest

The authors confirm that there are no conflicts of interest.

## Funding source

None declared.
